# Prevalence of Comorbidities, Overweight and Obesity in an International Sample of People with Multiple Sclerosis and Associations with Modifiable Lifestyle Factors

**DOI:** 10.1371/journal.pone.0148573

**Published:** 2016-02-05

**Authors:** Claudia Helena Marck, Sandra Leanne Neate, Keryn Louise Taylor, Tracey Joy Weiland, George Alexander Jelinek

**Affiliations:** Neuroepidemiology Unit, Melbourne School of Population and Global Health, The University of Melbourne, Melbourne, Australia; University of Oxford, UNITED KINGDOM

## Abstract

Multiple sclerosis (MS) is a chronic neurological disorder, often affecting young people. Comorbid disorders such as depression, anxiety and hypertension are common and can affect disease course, treatment, and quality of life (QOL) of people with MS (PwMS). The associations between comorbidities, body mass index (BMI) and health outcomes are not well studied in MS, although research shows most PwMS are overweight. Most data on the prevalence of comorbidities and obesity in PwMS comes from North American populations. This study describes the prevalence of comorbidities, overweight and obesity and associations with modifiable factors in an international sample of PwMS recruited online through social media, MS societies and websites. The online survey consisted of validated and researcher-devised instruments to assess self-reported health outcomes and lifestyle behaviors. Of the 2399 respondents, 22.5% were overweight, 19.4% were obese and 67.2% reported at least one comorbidity, with back pain (36.2%), depression (31.7%), anxiety (29.1%) and arthritis (13.7%) most prevalent and most limiting in daily activities. Obesity and most comorbid disorders were significantly more prevalent in North America. Obese participants were more likely to have comorbidities, especially diabetes (OR 4.8) and high blood pressure (OR 4.5) but also depression (OR 2.2). Being overweight, obese, or a former, or current smoker was associated with an increase in the number of comorbidities; while healthy diet, physical activity (borderline significant) and moderate alcohol consumption were associated with decreased number of comorbidities. Increasing number of comorbidities was related to worse QOL, increased odds of disability and prior relapse. Obese PwMS had higher odds of disability and lower QOL. The associations between BMI, comorbidities and health outcomes are likely to be bi-directional and associated with lifestyle behaviors. Preventing and treating comorbid disorders and obesity in PwMS is warranted, and advice regarding healthy and risky lifestyle may assist in improving health outcomes.

## Introduction

Multiple sclerosis (MS) is the most common serious neurological disorder in young people and is often progressively disabling. Recently there has been a growing interest in the influence of comorbidities on the health of people with MS (PwMS); comorbidities have been shown to be associated with increased hospitalization[1], rate of progression to disability [2, 3], and decreased quality of life (QoL)[4]. Comorbidities are associated with increased mortality risk in the general population as well as in PwMS[5]. PwMS may have specific risks for comorbid disorders due to side effects of pharmacological therapies[6] or due to underlying pathology. Further, adverse health behavior including being overweight and obese, smoking and sedentary behaviour, which are known risk factors for adverse health outcomes, are common in PwMS[7]. Additionally, PwMS may have an increased chance of being diagnosed with a comorbid disorder as they make more use of health services[8].

Marrie’s group recently completed a series of systematic reviews on the incidence and prevalence of comorbidities in PwMS worldwide and concluded that findings are inconsistent in Europe and North America, and very little data exist for other regions where MS is less prevalent[9]. The most prevalent comorbidities identified in their meta-analysis were depression, anxiety and hypertension [9]. The prevalence of obesity was not included in this work, but several studies have reported that the majority of people with MS are overweight or obese[7, 10–12]. While being overweight or obese increases the risk for MS and comorbid disorders, body mass index (BMI) does not seem to be a predictor of disability progression[10].

This study examined the prevalence of comorbidities, overweight, and obesity, and their associations with health outcomes in a large international cross-sectional sample of PwMS who self-enrolled in the Health Outcomes and Lifestyle Interventions in a Sample of people with Multiple Sclerosis (HOLISM) study[13].

## Methods

The methods of the HOLISM study have been described in detail elsewhere[13] but relevant details are summarised here. The study was approved by the Human Research Ethics Committee of St Vincent’s Hospital Melbourne (LRR 055/12).

### Participants

Participants were recruited online in 2012 using social media including Facebook and Twitter, MS society websites and newsletters and other websites and forums all over the world specifically for people with MS. Many of these media had a healthy lifestyle focus and most were in English. A hyperlink took people to a SurveyMonkey webpage detailing participant information where participants consented to participate in the study and confirmed that they were 18 years or older before entering the survey. Analyses were only performed for those completing the Self-administered Comorbidity Questionnaire (SCQ) and reporting a physician confirmed diagnosis of MS.

### Tools and instruments

This English-language survey took approximately 40 minutes to complete and comprised validated and researcher-devised or amended questionnaires. All data were self-reported. Several multiple choice items assessed demographic variables of age, gender, country of residence, height and weight. Country of residence was grouped into 4 categories to enable analysis: United States and Canada; Australia and New Zealand; Europe; and Other.

### Health outcome measures

Comorbidity was assessed using the SCQ [14], which is an efficient method of assessing the presence of comorbidities when medical record review is impractical, and whether the comorbid condition is treated and whether it limits daily activities. For the purpose of this study, two arthritic conditions were combined into one, and we added anxiety as a listed comorbid condition. The SCQ has previously been used to study PwMS[15] and correlates modestly with the Charlson Comorbidity Index.

Disability was measured by the Patient-Determined Disease Steps (PDDS), a self-administered surrogate tool for the commonly used clinician-administered Expanded Disability Status Scale (EDSS)[16]. For the purpose of data analysis, scores from 0 (normal) to 8 (bed bound) were collapsed into two categories: scores of 0–3 (being able to walk more than 25 feet without assistance) were categorized as no/mild disability; scores of >3 were categorized as moderate/severe disability.

Health related quality of life (HRQOL) was measured by the widely used and validated Multiple Sclerosis Quality Of Life-54 (MSQOL-54)[17], which consists of 54 items, including the 36-item Short Form Health Survey (SF-36), which generates two composite scores: the physical and mental health composites and 12 subscales.

Relapse rate was assessed by self-reporting of how many medically confirmed relapses participants with relapsing-remitting MS had had in the previous 12 months. Participants were categorized as having reported 1 or more relapses in the previous 12 months or no relapses.

### Modifiable factors

BMI was calculated by dividing weight (in kilograms) by height^2^ (in centimetres) and categorized according to World Health Organization (WHO) standards so that those with BMI below 18.5 were classed as underweight, BMI of 18.5 to 25 as normal, BMI of 25 to 30 as overweight and BMI of 30 and over as obese. Physical activity was categorized as low, moderate or high, as assessed with the International Physical Activity Questionnaire (IPAQ)[18], and diet was assessed using the Dietary Health Questionnaire (DHQ), on a 1–100 point scale, and has been previously described for this sample[19]. Alcohol consumption, tobacco smoking, meditation practice, omega-3 fatty acid and vitamin D supplementation were assessed using researcher-devised items and prevalence in this sample has also been previously been described[20–23]. Alcohol consumption was categorized as follows: low (<15g/week), moderate (up to 30g/day for females; up to 45g/day for men), or high for consumption above those amounts; and smoking status was self-reported. Participants were asked to select if they were currently using a disease modifying drug (including glatiramer acetate, interferons, natalizumab, alemtuzumab, daclizumab, rituximab, fingolimod, dimethyl fumarate, teriflunomide, cladribine, laquinimod, and azathioprine) as described in detail previously[24].

### Conceptual model

**Fig 1:**
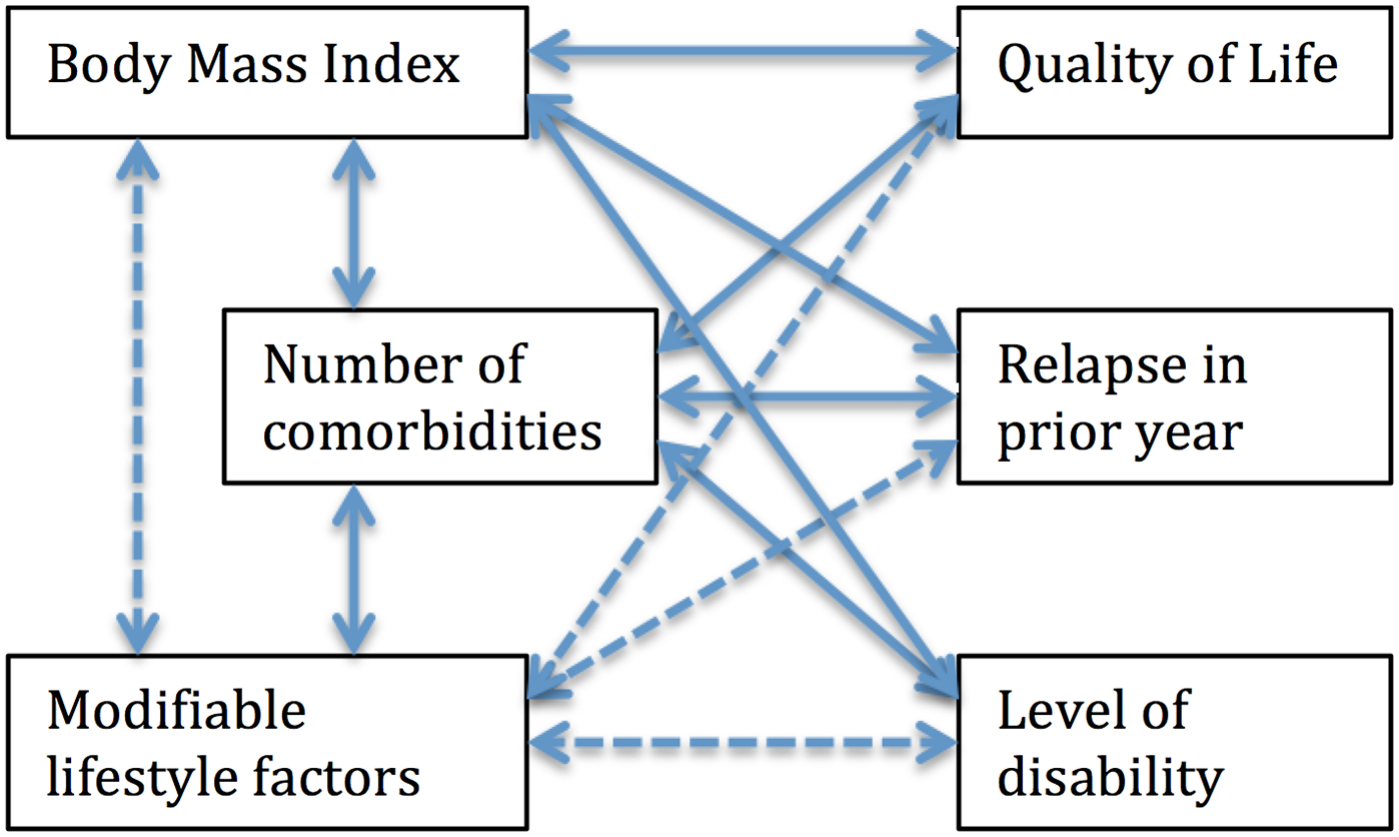
Associative conceptual model.

The associative conceptual model used to guide analysis is shown in Fig 1. Hypothesised associations between variables shown in this model were investigated, except for the associations depicted by dashed arrows, as these have been investigated separately [18–24].

### Data analysis

Data were analyzed using SPSS version 23.0 (IBM Corporation). Continuous data were reported using mean and 95% confidence interval (95% CI) or standard error (SE), or median (IQR) and categorical data using number (N) and percentage (%). Pearson’s Chi Square or Fisher’s exact test were used to compare categorical variables, with standardized adjusted residuals indicating over or underrepresentation of groups. Spearman’s rho was used for non-parametric correlation. Analysis of variance was used to compare continuous variables across three or more groups with LSD post-hoc testing or Games-Howell if there was homogeneity of variances. Kruskal Wallis testing was used for comparisons between groups if the variable was not normally distributed with Bonferroni adjustments applied to non-parametric post-hoc tests. Multiple regression (enter method) was used to identify independent variables associated with number of comorbidities and HRQOL. It was ensured that data satisfied the assumptions of normality, linearity, and homoscedasticity by plotting the studentized residuals against the unstandardized predicted values of the dependent variable and assessing the spread. Independence of residuals was assessed by checking the Durbin Watson statistic (values between 1 and 3 were accepted). Variance inflation factor <5 was used as the criterion for absence of multicollinearity. Only correlations of < .70 between independent variables were accepted as inspected from the correlation matrices. Binary logistic regression was used to identify independent variables associated with disability and relapse. Due to participant numbers being low in some regions of the world, regions were collapsed in 4 categories: United States (US) and Canada; Australia and New Zealand; Europe; and Other.

Alpha was set at 0.05 and two tailed tests of significance were used in all instances. Varying denominators are due to varying item completion.

## Results

The characteristics of the whole HOLISM sample have been described elsewhere[13]. Here we report data on the 2399 participants who completed the SCQ; 407 (17.7%) men and 1892 (82.3%) women, with an average age of 45.5 years (SD 10.6), diagnosed with MS for a median of 6 years (IQR 3–12), with most reporting having relapsing-remitting MS (1472, 61.6%).

**Fig 2:**
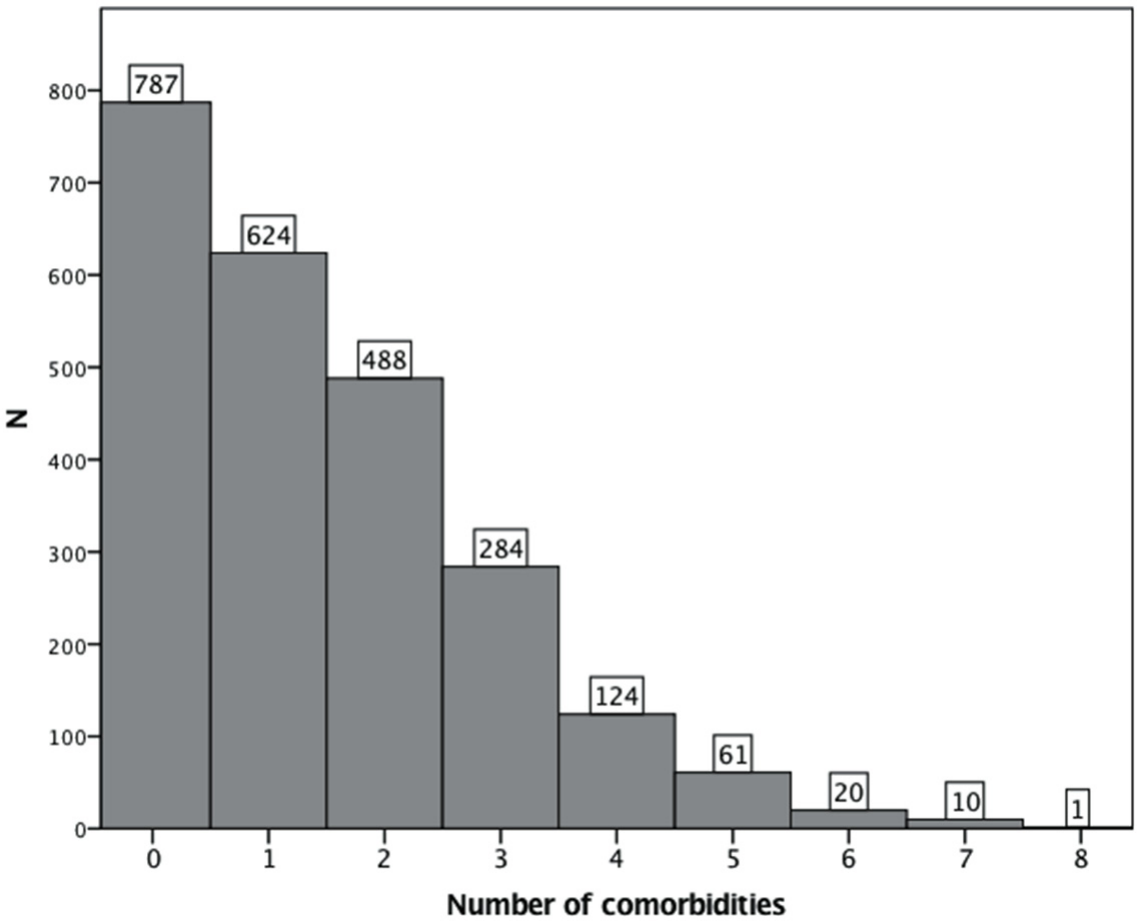
Frequency of comorbidities.

**Fig 3:**
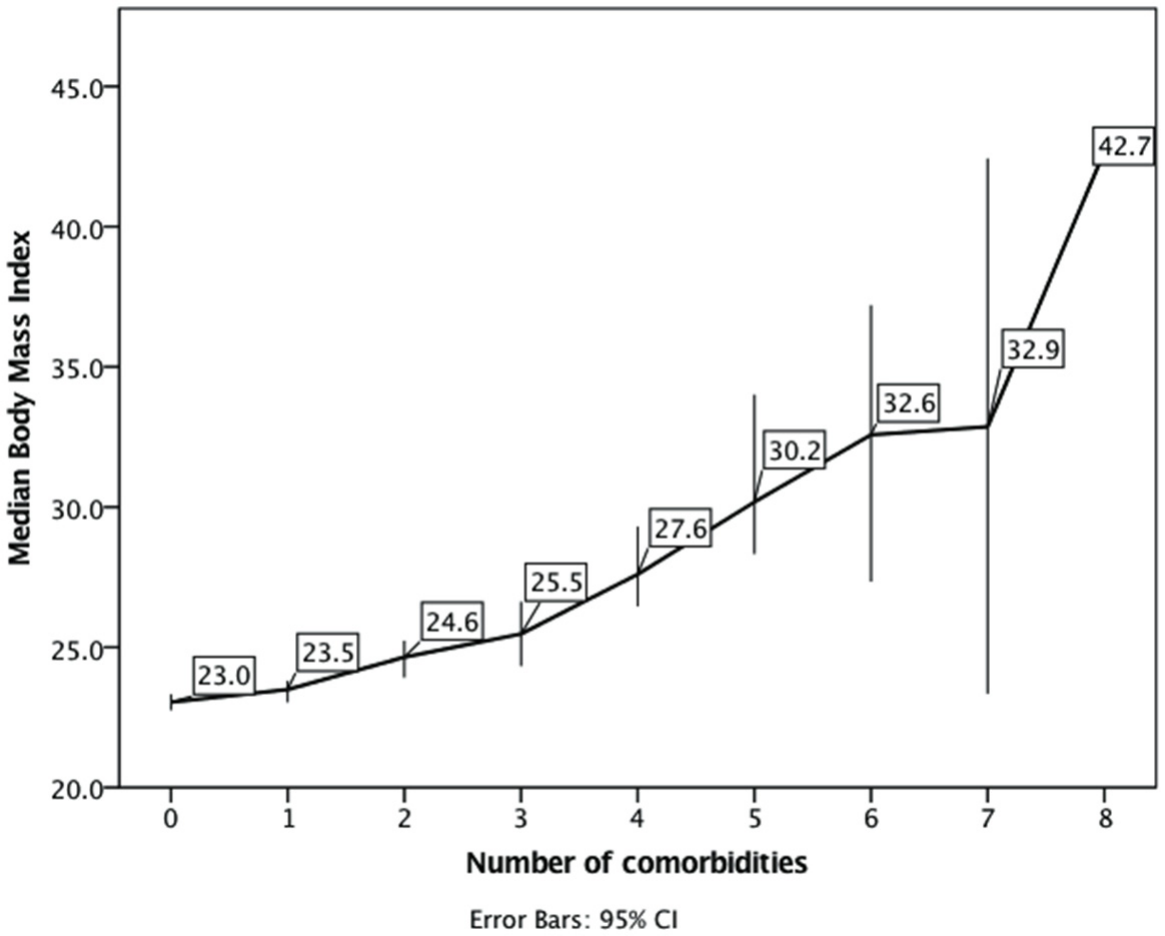
Association of comorbidities with body mass index.

Almost a third (787, 32.8%) reported no comorbidities; one person reported 8 (Fig 2), the median was 1.0 (IQR 0–2). Within this sample, 2366 (98.6%) participants completed data on height and weight. The median BMI was 24.0 (IQR 21.3–28.3); most (53.6% 95%CI 51.5–55.5) had a normal weight, 4.2% (95%CI 3.4–5.1) were underweight, 22.5% (95%CI 21.1–24.4) were overweight and 19.4% (95%CI 18.0–21.2) were obese. BMI was related to the number of comorbidities reported (Spearman’s rho = .23, p < .001) and the association between median BMI and number of comorbidities is shown in Fig 3.

The most commonly reported comorbidity, in more than a third of participants, was back pain, followed by depression and anxiety which were both reported by around 30% of PwMS (Table 1). The four most common comorbidities caused the greatest limitations to activities of daily life. For all comorbidities other than back pain, arthritis, and liver disease the majority were receiving treatment.

**Table 1:** Prevalence of comorbidities.

Comorbidities	Has the condition		Receives treatment*		Limits daily activity*	
	N	% (95% CI)	N	%	N	%
Back pain	869	36.2 (34.3–38.1)	381	43.8	454	52.2
Depression	760	31.7 (29.9–33.6)	531	69.9	302	39.7
Anxiety	699	29.1 (27.4–31.0)	351	50.2	300	42.9
Arthritis	328	13.7 (12.3–15.0)	119	36.3	165	50.3
High blood pressure	269	11.2 (9.8–12.5)	227	84.4	13	4.8
Anaemia or other blood disease	154	6.4 (5.5–7.4)	92	59.7	28	18.2
Ulcer or stomach disease	108	4.5 (3.7–5.3)	79	73.1	22	20.4
Diabetes	63	2.6 (2.0–3.3)	47	74.6	7	11.1
Lung disease	61	2.5 (1.9–3.2)	50	82.0	17	27.9
Heart Disease	54	2.3 (1.7–2.9)	37	68.5	12	22.2
Cancer	50	2.1 (1.5–2.6)	31	62.0	5	10.0
Kidney disease	21	0.9 (0.5–1.3)	12	57.1	8	38.1
Liver disease	15	0.6 (0.3–1.0)	5	33.3	2	13.3

Total N = 2399

*Percentage reflects proportion of those with the condition

There was a clear association between obesity and the number of comorbidities (p < .001); obese participants were significantly less likely to have no comorbidities (17.2% vs 36.7% of non-obese participants) and more likely to have 3 or more comorbidities compared to non-obese participants (38.0% vs 16.7%). Those with obesity were more likely, compared to those without obesity, to have back pain (OR 2.0; 95%CI 1.7–2.5, p < .001), depression (OR 2.2; 95%CI 1.8–2.7, p < .001), anxiety (OR 1.7; 95%CI 1.3–2.0, p < .001), arthritis (OR 2.3; 95%CI 1.8–3.0, p < .001), high blood pressure (OR 4.5; 95%CI 3.4–5.8, p < .001), anaemia or other blood diseases (OR 1.5; 95%CI 1.0–2.2, p = .035), ulcer or stomach disease (OR 2.9; 95%CI 1.9–4.3, p < .001), diabetes, (OR 4.8; 2.9–8.0, p < .001), heart disease (OR 2.1; 95%CI 1.2–3.8, p = .014), or liver disease (OR 4.8; 95%CI 1.7–12.3, p = .003), but not cancer, kidney or lung disease.

### Sociodemographics

Gender was associated with obesity (p < .001) and number of comorbidities (p < .001); with women more likely to be obese than men (21.3% vs 10.5%), to have any comorbidity (68.7% vs 59.0% of men), and also to have more than 3 comorbidities (22.1% vs 14.7% of men). There was no significant difference in age between obese and non-obese participants, but those with more comorbidities were significantly older (p < .001). Post-hoc testing showed significant differences (all p < .001) in age between those without comorbidities (43.6 years, 95%CI 42.8–44.3 years), 1 comorbidity (45.7 years, 95%CI 44.9–46.5 years), 2 comorbidities (46.7 years, 95%CI 45.7–48.3 years) and 3 or more comorbidities (47.4 years, 95%CI 46.5–48.3 years) as well as a difference between 1 and 3 or more comorbidities (p = .007). Table 2 shows that obesity and most comorbidities were significantly more prevalent in the US and Canada. Mean age was significantly different between regions with the highest mean age in Australia and New Zealand (47.1 years, SE .38), then US and Canada (45.8 years, SE .34), then Europe (43.9 years SE .43) and then Other (39.5 years, SE 1.20) (all post-hoc tests p < .01).

**Table 2:** Comorbidity and BMI according to region.

	Region								
	United States and Canada		Australia and New Zealand		Europe		Other		P-Value
	N	% (95%CI)	N	% (95%CI)	N	% (95%CI)	N	% (95%CI)	
Underweight	37	4.2 (3.0–5.6)	33	4.1 (2.8–5.6)	29	4.7 (3.2–6.5)	1	1.5 (0.0–4.6)	
Normal weight	**385**	**43.9**~ **(40.5–47.1)**	**461**	57.3* **(53.9–60.6)**	**384**	62.1* **(58.1–66.0)**	37	55.2 (43.5–67.2)	
Overweight	213	24.3 (21.5–27.0)	183	22.8 (19.8–25.4)	128	20.7 (17.5–24.1)	15	22.4 (12.7–32.8)	< .001
Obesity	**242**	27.6* **(24.7–30.7)**	**127**	**15.8**~ **(13.4–18.5)**	**77**	**12.5**~ **(9.9–15.0)**	14	20.9 (11.4–31.7)	
Back pain	**346**	39.0* **(35.8–42.2)**	289	35.4 (32.2–38.6)	**200**	**31.9**~ **(28.1–35.5)**	**34**	50.0* **(38.2–62.7)**	.003
Depression	**354**	39.9* **(36.6–43.1)**	**221**	**27.1**~ **(24.1–30.2)**	**158**	**25.2**~ **(21.7–28.5)**	27	39.7 (29.0–52.2)	< .001
Anxiety	**309**	34.8* **(31.5–38.0)**	**216**	**26.4**~ **(23.5–29.6)**	**148**	**23.6**~ **(20.0–27.1)**	26	38.2 (27.5–50.0)	< .001
Arthritis	**160**	18.0* **(15.4–20.5)**	107	13.1 (10.8–15.5)	**49**	**7.8**~ **(5.7–10.0)**	12	17.6 (8.8–27.1)	< .001
High blood pressure	**148**	16.7* **(14.3–19.2)**	78	9.5 (7.5–11.7)	**34**	**5.4**~ **(3.6–7.3)**	9	13.2 (5.8–22.4)	< .001
Anaemia or other blood disease	57	6.4 (5.0–8.0)	53	6.5 (4.8–8.2)	38	6.1 (4.3–8.0)	6	8.8 (2.9–16.2)	.851
Ulcer or stomach disease	**51**	5.7* **(4.2–7.4)**	**24**	**2.9**~ **(1.9–4.1)**	25	4.0 (2.7–5.5)	**8**	11.8* **(4.4–20.6)**	.001
Diabetes	**36**	4.1* **(2.8–5.4)**	19	2.3 (1.4–3.4)	**5**	**0.8**~ **(0.2–1.6)**	3	4.4 (0.0–10.3)	.001
Lung disease	28	3.2 (2.1–4.4)	18	2.2 (1.2–3.3)	13	2.1 (1.1–3.2)	2	2.9 (0.0–7.4)	.507
Heart Disease	**31**	3.5* **(2.3–4.8)**	**12**	**1.5**~ **(0.7–2.3)**	**7**	**1.1**~ **(0.3–2.1)**	**4**	5.9* **(1.4–11.9)**	< .001
Cancer	23	2.6 (1.6–3.6)	14	1.7 (0.9–2.6)	12	1.9 (0.8–3.2)	1	1.5 (0.0–4.6)	.596
Kidney disease	12	1.4 (0.7–2.1)	5	0.6 (0.1–1.2)	3	0.5 (0.0–1.1)	1	1.5 (0.0–4.5)	.221
Liver disease #	6	0.7 (0.2–1.2)	4	0.5 (0.1–1.0)	3	0.5 (0.0–1.1)	2	2.9 (0.0–7.5)	.095

* Significantly overrepresented

^~^ significantly underrepresented

^#^ Chi-square not reliable due to low expected N in >20% of the cells. N = 2366 for BMI and N = 2399 for all comorbidities

### Modifiable lifestyle factors

Table 3 shows unadjusted and adjusted regression parameters associated with the number of comorbidities for each modifiable lifestyle factor variable. The variables disease-modifying drugs and meditation did not show significant associations and were therefore not included in the adjusted regression model. A linear regression model including age, gender, BMI, physical activity, diet, alcohol consumption, smoking status, vitamin D and omega-3 supplementation explained 11.7% of the variance in the number of comorbidities. Being overweight, obese, a former or current smoker were associated with an increase in the number of comorbidities; while healthy diet and moderate alcohol consumption were associated with decreased number of comorbidities. A high level of physical activity was borderline significantly associated with a decrease in comorbidity (p = .054).

**Table 3:** Associations between modifiable lifestyle factors and number of comorbidities,

	Unadjusted regression				Adjusted regression *			
	B	Sig.	95% CI		B	Sig.	95% CI	
Body Mass Index								
Underweight	0.06	0.691	-0.24	0.37	-0.05	0.736	-0.35	0.24
Overweight	**0.35**	**< .001**	0.20	0.51	**0.21**	**0.007**	0.06	0.36
Obese	**1.07**	**< .001**	0.91	1.24	**0.81**	**< .001**	0.64	0.98
Normal	#	.	.	.	#	.	.	.
Physical activity								
High	**-0.49**	**< .001**	-0.65	-0.33	-0.15	0.054	-0.31	0.00
Moderate	**-0.33**	**< .001**	-0.48	-0.17	-0.05	0.489	-0.20	0.09
Low	#	.	.	.	#	.	.	.
Diet score (1–100)	**-0.02**	**< .001**	-0.03	-0.02	**-0.01**	**0.003**	-0.02	0.00
Alcohol consumption								
High	-0.15	0.694	-0.90	0.60	-0.33	0.355	-1.03	0.37
Moderate	**-0.44**	**< .001**	-0.58	-0.31	**-0.33**	**< .001**	-0.46	-0.20
Low	#	.	.	.	#	.	.	.
Smoking status								
Current	**0.86**	**< .001**	0.65	1.06	**0.71**	**< .001**	0.51	0.91
Former	**0.26**	**< .001**	0.13	0.40	**0.21**	**0.002**	0.08	0.34
Never	#	.	.	.	#	.	.	.
Vitamin D supplementation								
>5000IU	**-0.40**	**< .001**	-0.61	-0.19	-0.08	0.445	-0.29	0.13
2001-5000IU	**-0.41**	**< .001**	-0.60	-0.23	-0.10	0.283	-0.30	0.09
1-2000IU	-0.17	0.098	-0.36	0.03	-0.07	0.445	-0.26	0.12
None	#	.	.	.	#	.	.	.
Omega-3 supplementation								
Yes	**-0.36**	**< .001**	-0.49	-0.22	-0.08	0.271	-0.22	0.06
No	#	.	.	.	#	.	.	.

* Covariates not displayed were age and gender

^#^ Reference category. N = 1864

### Outcomes measures

#### Disability

The sample consisted of 1212 (55.6%) people with mild or no disability (able to walk unassisted) and 967 (44.4%) people with moderate or severe disability. A binary logistic regression was carried out to assess the association between BMI, comorbidities and disability, while controlling for time since diagnosis, age and gender. The regression model was significant and showed that obese participants had 1.38 (1.07–1.78, p = .013) the odds of being in the moderate/severe disability category compared to those with normal BMI. Compared to having no comorbidities, the odds of being in the moderate/severe disability category for those with 1 comorbidity was 1.38 (95%CI 1.11–1.72 p = .003), for those with 2 comorbidities it was 1.59 (95% 1.18–2.15, p = .002), and for those with 3 or more comorbidities it was 1.89 (1.28–2.77, p < .001).

#### Health Related Quality of life (HRQOL)

The mean physical HRQOL score was 59.3 (95%CI 58.3–60.3) and the mean mental HRQOL score was 67.1 (95%CI 66.2–68.1). A multiple linear regression model predicting both the mental and physical HRQOL showed that an increasing number of comorbidities was associated with decreasing mental and physical HRQOL, and overweight or obesity was associated with lower mental and physical HRQOL, while controlling for age, gender and level of disability (Table 4).

**Table 4:** Health related quality of life regression model.

	Physical Health Related Quality of Life #			Mental Health Related Quality of Life *		
Parameter	B	P	95% CI	B	P	95% CI
Comorbidities						
None	16.3	<0.001	14.5–18.1	24.0	<0.001	21.8–26.3
1	11.5	<0.001	9.6–13.3	16.3	<0.001	14.0–18.6
2	6.3	<0.001	4.4–8.2	8.9	<0.001	6.5–11.3
3 or more	Reference			Reference		
Body Mass Index						
Underweight	0.6	0.728	-2.7–3.9	1.0	0.610	-2.9–4.9
Overweight	-2.5	0.002	-4.0–0.9	-2.7	0.005	-4.6–0.8
Obese	-5.5	<0.001	-7.2–3.9	-2.1	0.051	-4.1–0.0
Normal	Reference			Reference		

Covariates were age, gender, level of disability.

^#^ Adjusted R Squared = .61

* Adjusted R Squared = .32

#### Relapse

Of those with relapsing-remitting MS, 53.1% (703) did not experience a relapse in the previous year while 46.9% (620) experienced one or more relapses. A binary logistic regression was carried out to assess the association between BMI, comorbidities and relapse, while controlling for age and gender. The regression model was significant and showed that BMI was not a significant correlate, while having comorbidities was significantly associated with increased risk for relapse. Compared to having no comorbidities, the odds of having had a relapse for those with 1 comorbidity was 1.68 (95%CI 1.30–2.18, p < .001), for those with 2 comorbidities it was 1.66 (95% 1.14–2.42, p = .008), and those with 3 or more comorbidities it was 2.60 (1.56–4.31, p < .001)

## Discussion

### Body Mass Index

Our results from a large international sample of PwMS showed that overall, 22.5% were overweight and an additional 19.4% were obese. There is currently no consensus on whether the prevalence of obesity is higher among PwMS compared to the general population[2]. However, the prevalence of obesity in our sample is considerably lower than the prevalence of obesity in the general population, in 2014 of the US (32.6% of men and 34.7% of women), Canada (32.6% of men and 34.7% of women), United Kingdom (26.9% of men and 29.2% of women), New Zealand (27.7% of men and 30.8% of women) and Australia (28.4% of men and 28.8% of women)[25], where most of our participant reside. Within our sample, the prevalence of obesity was significantly higher in US and Canada (27.6%) compared to Europe (12.5%) and Australia and New Zealand (15.8%). North American data from 2006 from the North American Research Committee on Multiple Sclerosis (NARCOMS) study showed that 55.8% were overweight or obese[7], comparable to the 51.9% of overweight or obese PwMS from US and Canada in our sample. However, other studies from North America report prevalence rates above 60%[26, 27]. One Spanish study reported a 39% prevalence rate of overweight (they did not specify obese) PwMS, higher than the 33.2% reported by European PwMS in our sample[28]. The relatively low prevalence of overweight and obesity may be due to the nature of the study as we recruited people with MS from websites and other online sources of which many were promoting healthy lifestyle, to complete a lifestyle survey.

Health outcomes for PwMS in our sample were better for those with lower BMI. Our data showed that overweight and obese PwMS reported lower mental and physical health HRQOL compared to those with normal weight while controlling for age, gender and level of disability and number of comorbidities. However, only obesity was associated with a clinically significant decrease of >5 points in physical HRQOL. A very small proportion in our sample was underweight (4.2%), and there were no differences in HRQOL from those with normal weight. Spanish data has shown that overweight PwMS had lower general and mental health scores compared to those with normal weight, however did not distinguish between overweight and obese, and found no differences in other quality of life scales of the SF-36, possibly due to the small number of people in the study[28]. Another study showed that obese PwMS scored lower on several QOL domains compared with non-obese PwMS but this study did not distinguish between overweight and normal weight[29]. In the general population, both underweight and obese people report lower QOL scores[30].

In our sample, BMI was significantly related to levels of disability, with obese participants 1.4 times more likely to have moderate/severe disability while controlling for age, gender, time since diagnosis and number of comorbidities. This association has been shown previously[31] but was not found in other studies[7, 10]. Current BMI was not associated with having had a relapse in the year prior to the study, similar to another study that did not find an association between BMI and hazard of relapse[32]. There may be a bi-directional association between obesity and health outcomes, and prospective and longitudinal data are needed to further assess this association[2].

### Comorbidities

There was a clear association between BMI and number of comorbidities, in line with expectations[33]. Obese participants were more likely to report comorbidities, with odds ratios up to 4.8 (diabetes). More than two thirds of PwMS reported at least one comorbid disorder, similar to previous North America data [34]. Women were more likely to report any comorbidity, while North American data that did not include mental comorbidities have previously shown women are less likely to have physical comorbid disorders[34]. The association found between gender and comorbidities may be mediated by BMI, as women were also more likely to be obese in our sample.

The most commonly reported comorbidity was back pain. Back pain is not commonly specifically studied in MS, but general pain and muscle or joint problems are common in PwMS (over 50%)[4]. Both depression (31.7%) and anxiety (29.1%) were common and more prevalent in our study than in a recent systematic review by Marrie et al. (summary prevalence of 23.7% and 21.9% respectively)[35]. Conversely, a recent summary prevalence estimate of hypertension of 18.6%[36], was higher than the 11.2% reported here. Arthritis was reported by a recent review to range between 3.0–26%[37], here reported to be 13.7%. Lung disease has been recently reported to be reasonably common, with a summary prevalence of 10%, but here only reported in our sample to be 2.5%[37]. Diabetes, here reported in 2.6% of PwMS, has been reported by several studies with inconsistent prevalence rates ranging between 0 and 27%[36]. Cancer, heart, kidney and liver disease were reported by less than 5%, in line with previous data[36–38]. Similarly, the prevalence of ulcer or stomach disease in our study (4.5%) was within a recently reported (albeit wide) range (1.8–18.4%)[37]. Discrepancies between prevalence in comorbidities may be caused by the international nature of our sample, whereas many other studies included mostly PwMS from Canada and the US[9]. The majority of participants received treatment for their comorbidities, except for back pain (44%), arthritis (36%) and liver disease (38%). The most common comorbidities: back pain, depression, anxiety and arthritis, were also the most limiting in daily activities.

Mental and physical health-related HRQOL scores decreased with increasing number of comorbidities, in line with findings from NARCOMS[39] and in other chronic diseases[40, 41]. Further, among those with relapsing-remitting MS, increasing number of comorbidities was associated with higher odds of having had a relapse in the year prior to the study, while controlling for BMI, age and gender. An increasing number of comorbidities was also associated with increasing odds to have moderate/severe disability, adjusted for age, time since diagnosis, gender and BMI. In our sample, prevalence of obesity, and most comorbidities was higher in PwMS residing in US and Canada compared to other regions in the world.

The relationship between weight, comorbidities and MS health outcomes is complex and difficult to disentangle and is likely to be affected by adverse health behaviors. Adjusted regression analysis showed that being overweight, obese or a former or current smoker were associated with an increase in the number of comorbidities; while healthy diet, physical activity (borderline significant) and moderate alcohol consumption were associated with decreased number of comorbidities. While we are unable to draw conclusions on the temporality or causality of these associations, due to the design of the study, these results suggest that further studies should assess these variables over time to elucidate causality. These relationships between healthy lifestyle, obesity and comorbidities may be expected, however, the impact of increasing risk of comorbidities may be of greater significance in PwMS who are already at risk of developing progressive physical disability. And while obesity and comorbidities including back pain, depression and anxiety may lead directly to decreased quality of life, obesity may also interfere with health and wellbeing indirectly through decreased physical activity[42] and quality of sleep[43]. Preventing or treating comorbidities and obesity in PwMS should be an important goal in MS management[44]. Advice regarding healthy lifestyle including diet[19], smoking[20, 45], and physical activity[18, 42] may be helpful in decreasing the risk for comorbidity, as well as improving health and wellbeing in its own right[46].

### Limitations

All data were self-reported and it is therefore not possible to assess accuracy or reliability. Measures of waist circumference or fat distribution may have added accuracy but due to the international nature of the study were not feasible. In another study from this sample, 19.3% screened positive for risk for depression as measured by the PHQ-2[47]. Here, 31.7% (760) reported depression including 9.5% (229) who are not currently treated for depression, 12.5% of the entire sample (302) reported that depression limits their daily activity, which indicates that for most, treatment seems effective and they may therefore currently not screen positive on the risk for depression. For some comorbidities, it can be difficult to distinguish between MS symptoms and a comorbidity. Participants in this study were English speaking and with the ability to complete this survey online, and most were women between the ages of 38 and 53 years. This sample may be less representative of the heterogeneous spectrum of PwMS, it may therefore not be generalizable to all PwMS, despite the size of the sample and the variety of backgrounds of participants. Due to the number of countries, we grouped these together in 4 broad regions for the purpose of analysis, but we recognize that variability may exist between countries grouped together. Due to the cross-sectional design of the study, and the complex interactions between the factors assessed, we cannot infer causality or temporality. This is further complicated by the different time frames used in the measures included in the survey. Relapses were assessed for the past 12 months and exercise over the previous 7 days, while only current comorbid disorders were assessed.

## Conclusion

In a large international sample of PwMS, those who were overweight or obese and had multiple comorbidities had worse health outcomes. Our study design precludes determination of causality, however there were clear associations of increasing BMI and increasing number of comorbidities with higher odds for disability and prior relapse and lower HRQOL. Similarly, lifestyle factors, including diet, smoking, and physical activity were associated with number of comorbidities. Further research, particularly longitudinal data, is required to better elucidate direction of causality. However, while further research is undertaken, preventing and treating comorbid disorders and obesity in PwMS is warranted especially in PwMS from North America and Canada where obesity and comorbidities were significantly more prevalent.
